# Biomarkers and their potential for detecting livestock plant poisonings in Western North America

**DOI:** 10.3389/fvets.2023.1104702

**Published:** 2023-02-22

**Authors:** Benedict T. Green, Kevin D. Welch, Stephen T. Lee, Clinton A. Stonecipher, Dale R. Gardner, Bryan L. Stegelmeier, T. Zane Davis, Daniel Cook

**Affiliations:** Poisonous Plant Research Laboratory, United States Department of Agriculture, Agricultural Research Service, Logan, UT, United States

**Keywords:** larkspurs, lupine (*Lupinus* spp.), water hemlock, swainsonine, selenium toxicity in animals, pyrrolizidine alkaloid (PA), Plants poisonous to livestock, ear wax

## Abstract

The United States National Cancer Institute defines a biomarker as: “A biological molecule found in blood, other body fluids, or tissues that is a sign of a normal or abnormal process, or of a condition or disease.” In Veterinary Medicine, biomarkers associated with plant poisonings of livestock have great utility. Since grazing livestock poisoned by toxic plants are often found dead, biomarkers of plant poisoning allow for a more rapid postmortem diagnosis and response to prevent further deaths. The presence and concentration of toxins in poisonous plants are biomarkers of risk for livestock poisoning that can be measured by the chemical analysis of plant material. More difficult is, the detection of plant toxins or biomarkers in biological samples from intoxicated or deceased animals. The purpose of this article is to review potential biomarkers of plant poisoning in grazing livestock in the Western North America including recently investigated non-invasive sampling techniques. Plants discussed include larkspur, lupine, water hemlock, swainsonine-containing plants, selenium-containing plants, and pyrrolizidine alkaloid containing plants. Other factors such as animal age and sex that affect plant biomarker concentrations *in vivo* are also discussed.

## Introduction

Plant poisonings of livestock are often a complex combination of plant and animal interactions making a definitive diagnosis challenging. Biomarkers that are accurate indicators of plant intoxications of livestock can be of great utility in managing plant poisonings. Biomarkers have been defined by the National Cancer Institute of the United States National Institutes of Health as: “A biological molecule found in blood, other body fluids, or tissues that is a sign of a normal or abnormal process, or of a condition or disease…”.^1^ The identification of biomarkers associated with poisonous plants will become increasingly important as climatic conditions have a dominant influence on vegetation (1) and poisonous plant problems tend to increase during times of decreased rainfall, when desirable forage becomes unavailable. Currently, there is a drought across Western North America, and livestock consumption of poisonous plants such as lupines, locoweeds, or larkspurs, which stay green longer into the dry part of the growing season, are likely to increase.

It is rare that plant toxins are acutely toxic, and that any exposure can result in fatal poisonings. In Western North America, livestock extensively graze on large pastures that can contain more than one poisonous plant (for example, larkspur and lupine). The presence of a sub-clinical concentration of one or more plant toxin does not indicate toxicity. In this context, identifying such plant toxins which are specific and sensitive biomarkers would be definitive evidence of poisoning. However, poisonous plants of Western North America are not that poisonous and identifying plant toxins only indicates exposure or ingestion, often subclinical in nature. A likely diagnosis would require associating such plant toxin biomarkers with associated with supporting clinical signs, biochemical changes and microscopic changes to obtain a more definitive diagnosis.

Plant toxins can be a variety of compounds including alkaloids (ammodendrine etc.), alcohols (cicutoxin) or elements (selenium). These types of plant biomarkers can be measured by the chemical analysis of plant material to yield a toxin concentration in plant material. Conversely, for livestock poisonings, animal biomarkers in biological samples from intoxicated or deceased animals are more difficult to identify. As a result, animal biomarkers as an indicator of plant poisonings are typically limited to the presence or absence of plant toxins or their metabolites in serum or tissues of poisoned animals. As with any livestock poisoning, there are five main factors to consider when investigating deaths that are suspected to be due to poisonous plants: 1: The poisonous plant must be present in the environment; the animal has access to it; and there is evidence that the poisonous plant has been consumed. 2: The toxin must be present in the plant material. 3: Plant material, toxins or toxin metabolites should be present in the ingesta (rumen/stomach/intestines content), and in biological samples from the intoxicated animal. 4: The clinical signs of the poisoning observed in the poisoned animal are known to be associated with that plant. 5: Other less specific biomarker(s) of poisoning such as hematology, serum biochemical, or tissue lesions are consistent with the suspected poisonous plant. Even if the five factors of plant poisonings have been satisfied, a dead animal is often found in a decomposed state precluding obtaining supporting clinical or postmortem support. The large pastures where livestock graze can have multiple toxic plant species present within the plant community making it difficult to determine the cause of the poisoning. A useful biomarker should be stable in a dead animal and easily detected in samples obtained during necropsy. Once identified as the cause of death, the responsible plant population could be fenced off from grazing livestock, potentially treated with a herbicide, and veterinary care initiated for the surviving animals. Toxic plants often associated with livestock losses in the Western North America include larkspur, lupine, water hemlock, swainsonine-containing plants, selenium accumulating plants and pyrrolizidine alkaloid containing plants. Many toxic plants can be nutritious to grazing livestock if safe quantities are consumed such that the toxins can be eliminated before causing intoxication. Poisoning by these plants occurs when animals eat too much, too fast, to cause acute poisoning or graze a poisonous plant over extended periods of time to cause chronic poisonings. This review will provide a summary of current knowledge of six significant poisonous plants and the plant and animal biomarkers most often associated with both acute and chronic poisonings from these plants in Western North America.

## Noninvasive specimens for plant toxin/biomarker analysis

One challenge of diagnosing livestock plant intoxications is the collection of biological samples from poisoned animals. Traditional specimens for analysis of plant toxins include samples such as serum, liver, or rumen contents (2). However, there are several other biological samples including hair, oral fluid, earwax, and nasal mucus that have, more recently, been evaluated as specimens for biomarker analysis in determining livestock consumption of poisonous plants. Hair and earwax are particularly useful samples because they can be collected from dead and live animals. This allows for the comparison of samples from lethally poisoned cases to that of other animals in a single location to document exposure to a poisonous plant. In other poisonings, oral fluid, and nasal mucus along with hair and earwax can be collected from non-lethally poisoned animals to estimate the amount of toxic plant exposure and to determine which animals may need veterinary care. Hair has been analyzed in cases of selenium toxicosis in horses (3). In this example, hair from the horse's manes and tails were used to identify chronic selenium poisoning in three of four horses grazed on the same pasture. This pasture had plants containing large amounts of selenium (e.g., watercress containing 127 μg/g selenium) and the animals had clinical signs of intoxication that included hoof lesions and lameness. Other investigators have measured fluoroacetate in earwax of cows that were experimentally administered sub-lethal doses of *Palicourea marcgravii*, a plant that causes sudden death syndrome in cattle, sheep, and goats in Brazil (4–6). Recently, earwax, hair, oral fluid, and nasal mucus were evaluated as noninvasive specimens to determine livestock exposure to poisonous larkspur plants and to toxic and teratogenic lupine plants (7). Lee et al. (7) also demonstrated that lupine alkaloids could be detected in the earwax and hair of cattle over a 30-day period and that lupine alkaloids could be detected in the oral fluid and nasal mucus of cattle over an 8-day time after receiving a single oral dose of lupine plant material. Lupine alkaloids were also detected in the earwax of cattle after a summer of grazing lupine-infested rangelands suggesting that plant alkaloids can persist in earwax months after exposure. Lastly, Stonecipher et al. (8) reported that larkspur alkaloids could be detected in the earwax, oral fluid, and nasal mucus of cattle over a 5-day period after receiving a single oral dose of larkspur plant. This suggests that noninvasive sampling can be used to detect plant alkaloids and to sample and manage intoxicated livestock more easily.

## Larkspurs (*Delphinium* spp.)

Larkspurs (*Delphinium* spp.) are a problem in North America due to their toxicity in grazing cattle and are much less toxic to other grazing ruminants such as goats (9, 10). There are at least 60 species of wild larkspurs (tall, low, and plains larkspurs) (11–16). Peak larkspur toxicity coincides with maximum productivity in areas where cattle are grazed, which, as a result, reduces the profitability of these rangelands. Larkspur-containing rangelands very productive and the loss of grazing on these rangelands is a significant economic impact to cattle producers in Western North America.

Larkspur toxicity in cattle is due to the pharmacological antagonism of nicotinic acetylcholine receptors (nAChR) at neuromuscular junctions by larkspur alkaloids to prevent the binding to and activation of the receptor by acetylcholine (17–22). The clinical signs of larkspur poisoning are due to the blockade of nAChR by larkspur alkaloids and include resistance to exercise, muscle weakness, lack of coordination, rapid heart rate, sternal recumbency followed by lateral recumbency (i.e., unable to maintain an upright posture even when lying down), bloating, and death. It is also speculated that larkspur alkaloids block vagal cholinergic neurotransmission to inhibit the function of esophageal smooth muscle (23) which interferes with eructation to cause bloat in cattle.

**Figure 1:**
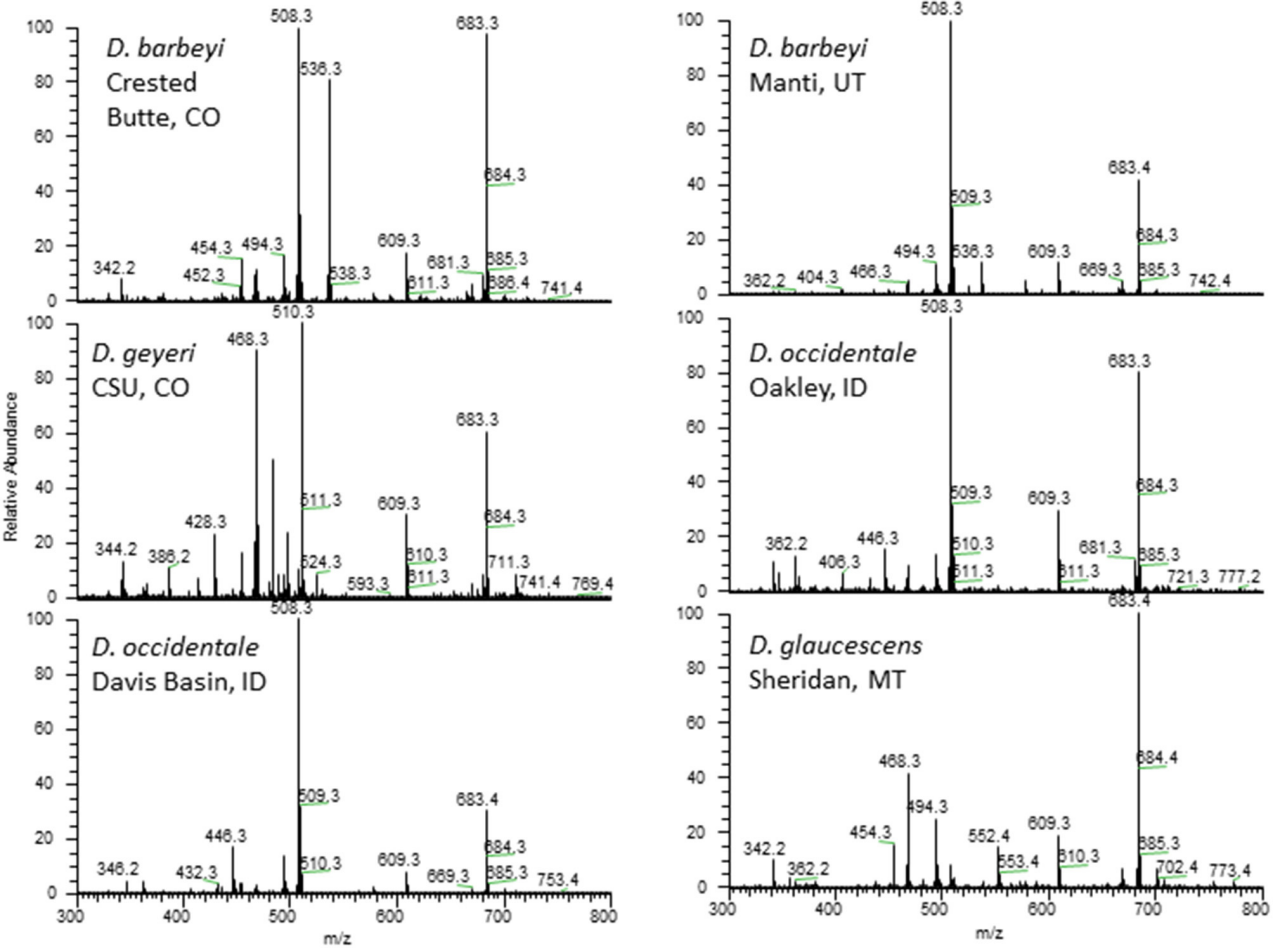
Electrospray mass spectra of 6 collections of *Delphinium*. Peaks of note include deltaline (MH^+^ = 508), methyllycaconitine (MH^+^ = 683), 14-acetylbrowniine (MH^+^ = 510), and 14-acetyldictyocarpine (MH^+^ = 536) (27).

**Figure 2:**
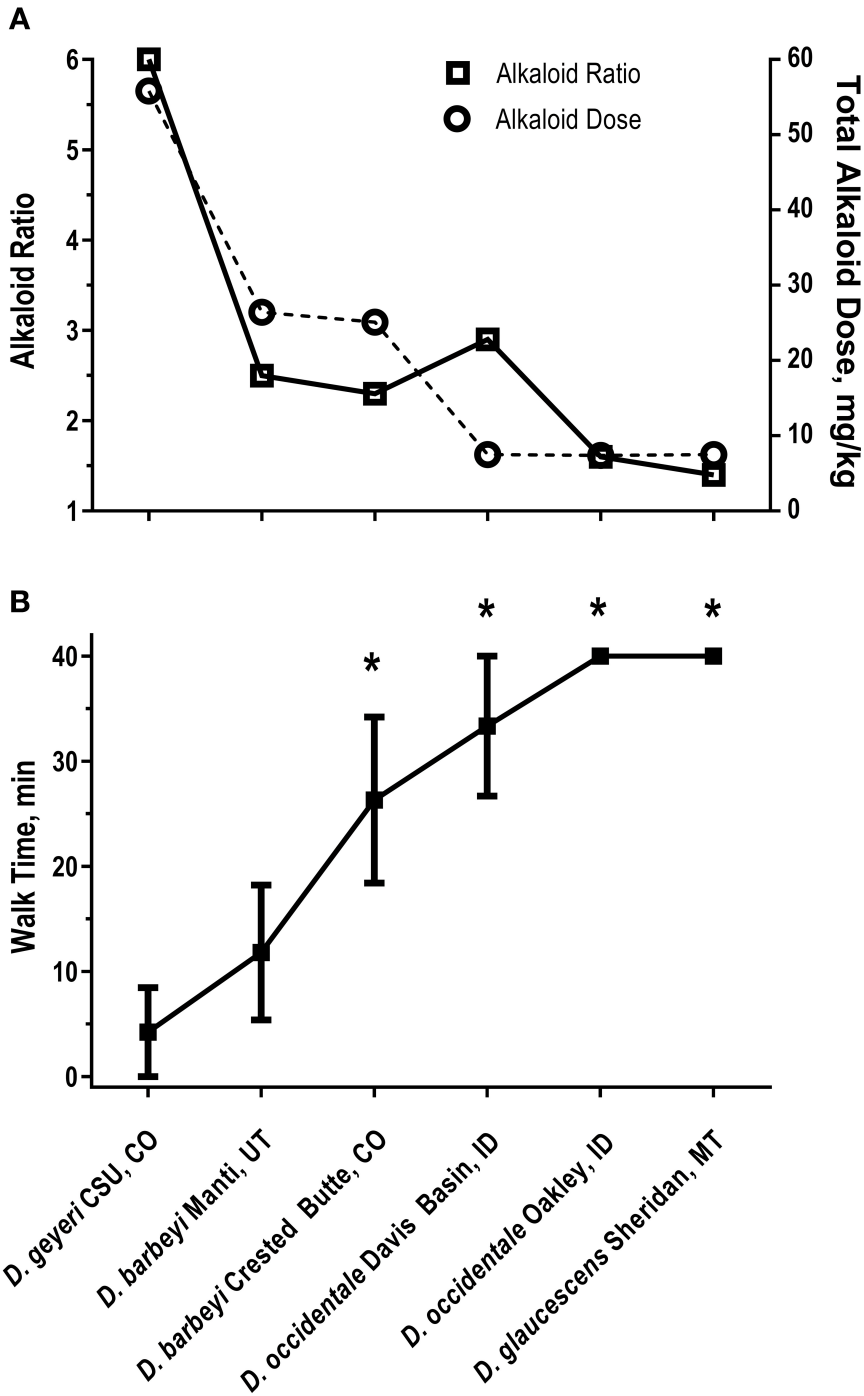
Alkaloid ratios and dose **(A)** and walk times **(B)** in six heifers dosed with larkspur (27). **(A)** Non-MSAL-type alkaloid to MSAL-type alkaloid ratios on the left y axis and total alkaloid dose on the right y axis. Walk times (min) were related to total alkaloid dose (*r* = −0.91 *P* = 0.0045) and alkaloid ratio (*r* = −0.80, *P* = 0.0258). **(B)** The mean ± SE of Angus heifer walk times 24 h after receiving a fixed dose of MSAL-type alkaloids (7.5 mg/kg *N*-(methylsuccinimido) anthranoyllycoctonine type alkaloids). The six plant collections dosed to the heifers were: *D. geyeri* CSU, CO (CSU, CO); *D. barbeyi* Manti, UT (Manti, UT); *D. barbeyi* Crested Butte, CO (Crested Butte, CO); *D. occidentale*, Davis Basin, ID (Davis Basin, ID); *D. occidentale* Oakley, ID (Oakley, ID); *D. glaucescens* Sheridan, MT (Sheridan, MT). *P < 0.05 vs. *D. barbeyi* Manti, UT.

Norditerpenoid alkaloids are the plant alkaloids associated with larkspur toxicity in cattle. These alkaloids include the more toxic *N*-(methylsuccinimido) anthranoyllycoctonine (MSAL) type like methyllycaconitine (MLA) and the less toxic 7,8-methylenedioxylycoctonine (non-MSAL) like deltaline (24–26). Samples to be analyzed for the measurement of larkspur alkaloids are typically analyzed by high performance liquid chromatography mass spectrometry (HPLC-MS) or Fourier-transform infrared spectroscopy (FTIR). For example, norditerpenoid alkaloid compositions of *Delphinium* species and populations may be variable as shown in Figure 1 which depicts the electrospray mass spectra of six different larkspur collections. Specifically, the relative abundance of MLA (MH^+^ = 683) varies between the six larkspur collections. MLA is a significant larkspur alkaloid because it is most often the MSAL-type alkaloid responsible for toxicity to cattle. It has been well documented that some species/populations contain greater concentrations of toxic MSAL-type alkaloids (28–33), while other larkspur species/populations contain mostly non-MSAL-type alkaloids (21, 30, 31, 34–36). The differences in toxicity between MSAL- and non-MSAL-type alkaloids in cattle have been shown with exercised-based assays that use walking to elicit clinical signs of intoxication in cattle (typical doses are 7–8 mg/kg MSAL-type alkaloids). Without exercise, these doses are innocuous to most cattle and the animals do not present clinical signs of intoxication which suggests that doses below 8 mg/kg result in sub-clinical serum alkaloid concentrations. Moreover, larkspur alkaloids are rapidly eliminated from the bodies of cattle, in Angus, serum MLA has a half-life of approximately 16 h (Table 1) which suggests that by 78 h most of the serum MLA has been eliminated. Based upon the results of larkspur-exercise experiments, the ratios of non-MSAL alkaloids to MSAL alkaloids can be used as larkspur alkaloid biomarkers to predict larkspur toxicity (27, 30, 35, 36). An example of this is depicted in Figure 2, as plant alkaloid ratios decrease, the length of the walk times after oral dosing with dried ground larkspur increase, which suggests that larkspurs with lower alkaloids ratios are less toxic to cattle. In summary, alkaloid profiles of *Delphinium* species are biomarkers that can be associated with a risk of poisoning to cattle. These results can then be used to predict risk of intoxication to cattle based upon the ratios of alkaloids present in the plant.

**Table 1:** Serum MLA toxicokinetic data for in Angus and Holstein orally dosed with 2.7 mg/kg MLA in the form of dried ground low larkspur (*D. andersonii*^a^*)* (37).

**Cattle breed (*n*)**	**Tmax^b^ (h)**	**Cmax^c^ (ng/ml)**	**AUC^d^ (ng-h/ml)**	**E1/2^e^ (h)**
Angus, (5)	15 ± 6	119 ± 25	4,107 ± 1,371	15.6 ± 1.5
Holstein, (4)	12 ± 5	74 ± 15^*^	1,756 ± 364^*^	11.8 ± 0.5^*^

a D. andersonii contains the MSAL-type alkaloids nudicauline, 14-deacetylnudicauline, and geyerline in addition to MLA. The steers were orally dosed with 12 mg/kg total MSAL-type alkaloids.

b Tmax, describes the time of maximal serum alkaloid concentration.

c Cmax, describes the maximal serum alkaloid concentration.

d AUC, the area under the curve.

e E${}_{\frac{1}{2}}$, the elimination half-life.

* P < 0.05, toxicokinetic parameter of Angus vs. Holstein, t-test, Prism version 6.01.

### Animal poisonings by larkspur

Larkspur species in Western North America cause large economic losses from cattle deaths, increased management costs, and reduced utilization of pastures and rangelands (38, 39). Cattle responses to larkspur are variable and influenced by multiple factors such as breed, age, and sex described below. These factors affect the serum concentrations of larkspur alkaloid biomarkers in cattle. In addition to plant alkaloid concentrations, heart rate can also be used as a measure of larkspur intoxication in cattle, although, it is challenging to measure in a field setting.

There are differences between breeds of cattle and their ability to clear larkspur alkaloids from serum. It is well documented that larkspur alkaloids are rapidly cleared from the serum of cattle (9, 40–42). However, there are significant differences between the half-life (E_1/2_) and other toxicokinetic parameters for the clearance of MLA in Angus cattle (41) compared to Holstein cattle (42) (Table 1). These results suggest that the genetic background of a herd must be considered when responding to a poisoning because some cattle may clear larkspur alkaloids from their serum more slowly than others and as a result become more severely poisoned.

**Figure 3:**
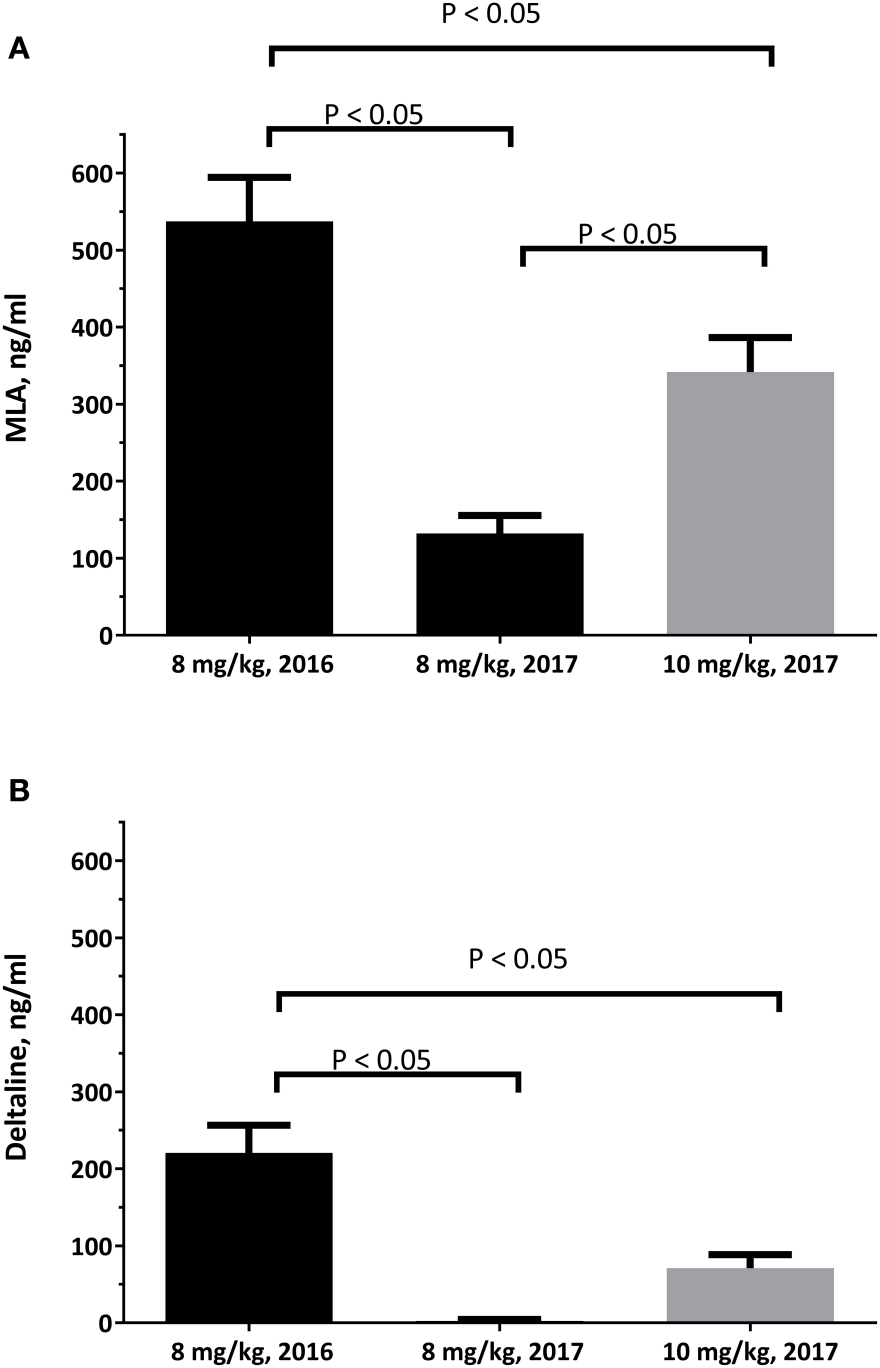
The serum concentrations of deltaline **(A)** and MLA **(B)** in 10 Angus steers at 24 hours after oral dosing. The serum samples were obtained just prior to the cattle being exercised during haltering and analyzed as described by Welch et al. (43). Statistical comparisons were made by ANOVA and Tukey's multiple comparisons test.

There are age-dependent differences associated with larkspur toxicity. These differences are thought to be due to changes in the ability of cattle to clear larkspur alkaloids from serum with age (Figure 3) (44). In laboratory-based experiments, serum deltaline concentrations changed from 221 ± 36 ng/ml at 24 h after dosing, in yearling cattle, to 3 ± 2 ng/ml at 24 h after dosing 1 year later (*P* < 0.0001 ANOVA). Serum MLA concentrations changed from 537 ± 57 ng/ml, in yearling cattle, to 132 ± 23 ng/ml 1 year later (*P* < 0.0001 ANOVA). As steers age, they can also clear a larger 10 mg/kg alkaloid dose more effectively than an 8 mg/kg dose administered as yearlings. Young cattle tend to consume more larkspur and are more severely affected by larkspur intoxication than older cattle (45, 46). This suggests that as cattle age, the serum concentrations of plant biomarkers can vary, and age must be considered when responding to larkspur poisonings. When yearling cattle are used as replacements, they should be carefully managed on larkspur-containing rangelands.

**Figure 4:**
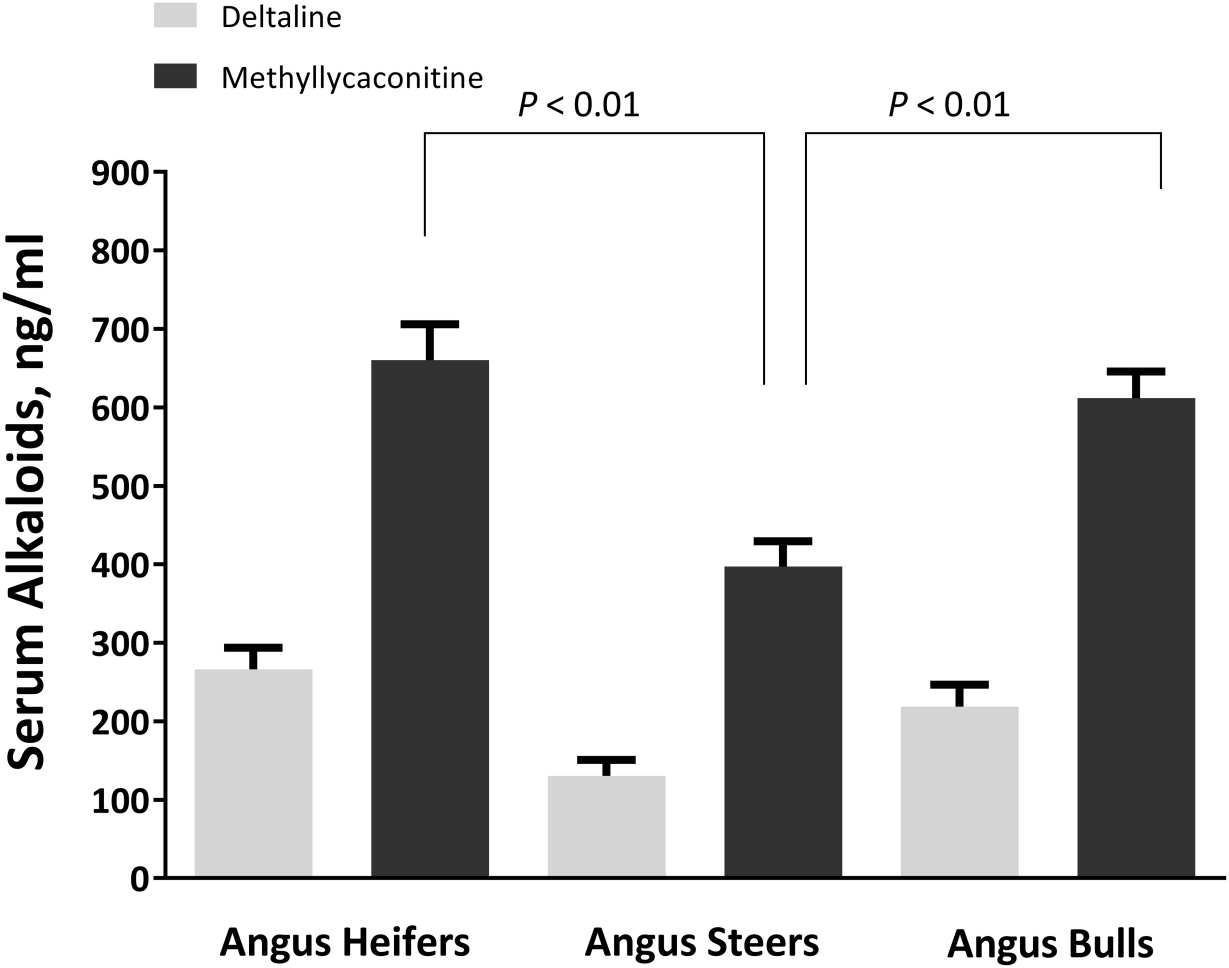
Serum alkaloid concentrations in heifers, steers, and bulls. The serum concentrations of deltaline and methyllycaconitine (MLA) (ng/mL) in 30 heifers, 23 steers, and 33 bulls at 24 h after oral dosing. Blood was collected at 24 h after dosing just prior to exercise. Chemical analysis of serum methyllycaconitine, and deltaline was performed as previously described (47). Serum alkaloid concentrations were compared using a one-way ANOVA with Tukey's multiple comparisons test (48).

**Figure 5:**
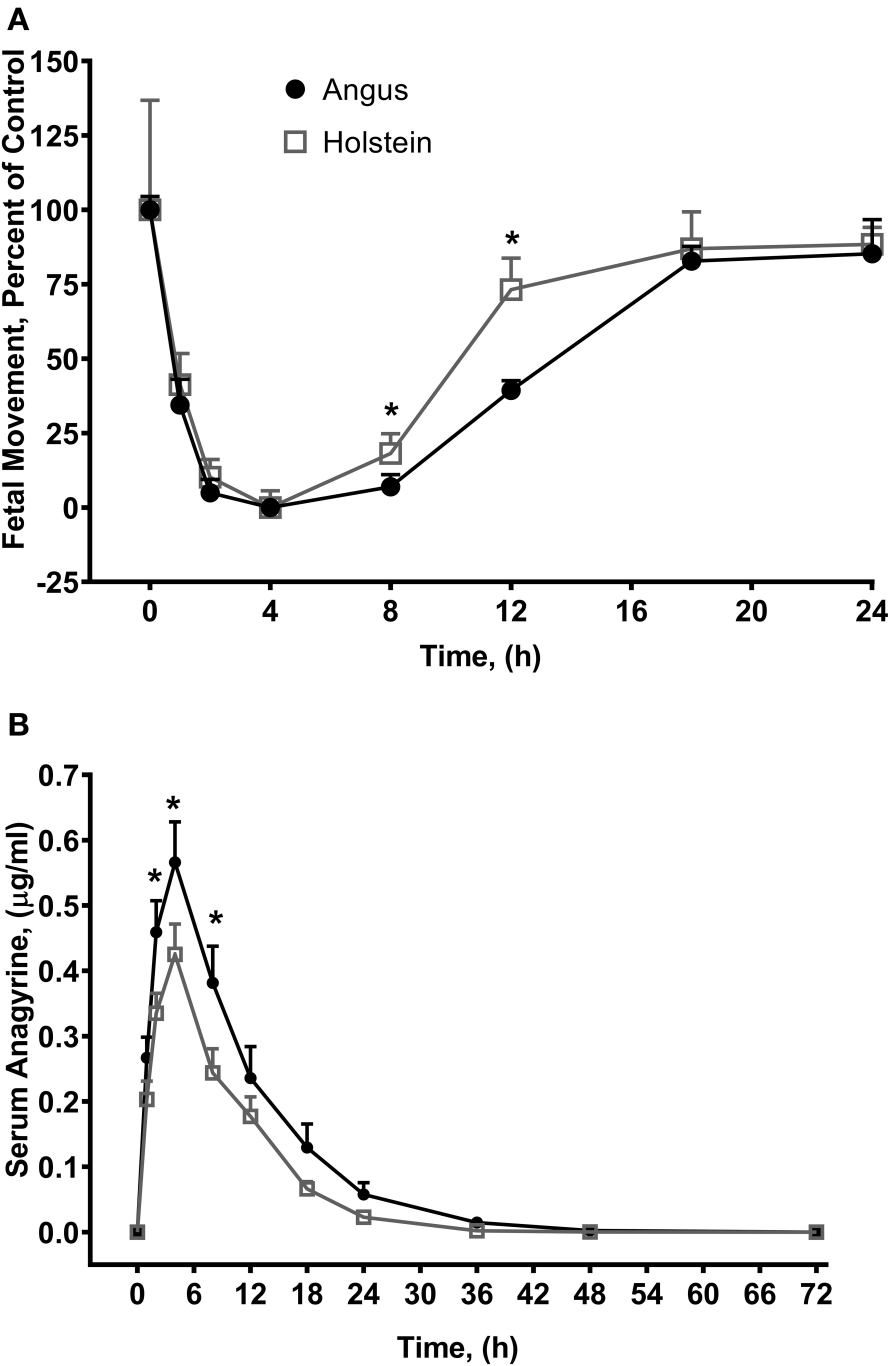
Fetal movements **(A)** and serum anagyrine concentrations **(B)** in Angus and Holstein heifers orally dosed with 1.1 g/kg body weight dried ground *L. leucophyllus* (49). Fetal movements were counted by transrectal ultrasound for 5 min and the number of fetal movements were normalized to time zero in the two breeds of cattle. Angus heifer fetuses averaged 42 ± 4 movements per five-minute sampling period and Holstein heifer fetuses averaged 31 ± 23 movements. Data represents the mean ± standard error. *P < 0.05 Angus (*n* = 6) vs. Holstein (*n* = 5) at each time point respectively (*t*-test). Serum anagyrine concentrations and fetal movements are correlated (Holstein, *P* < 0.0001; Angus, *P* < 0.0001).

Sex plays a role in the serum concentrations of larkspur alkaloid biomarkers (Figure 4) (48). When yearling heifers, bulls, and steers were dosed with 8 mg/kg MSAL-type alkaloids in the form of dry, finely ground larkspur; heifers and bulls had larger serum MLA concentrations than did steers (Figure 5). Serum deltaline concentrations were not significantly different between the three groups of cattle. Interestingly, heifers exercised the least after dosing with larkspur even though their serum alkaloid concentrations were not different from bulls. When adjusted for censoring due to the experimental methods used, heifers were only able to exercise for −8.89 ± 3.90 min compared to 13.18 ± 3.68 min for bulls and 15.93 ± 2.72 min for steers. These results suggest that sex must be considered when evaluating the serum concentrations of larkspur alkaloid biomarkers. Replacement heifers should not be placed on pastures with large amounts of toxic larkspur.

Finally, heart rate can be used as a measure of larkspur intoxication in cattle, and although heart rate is not a biomarker as defined by the National Cancer Institute it is consistently elevated in cattle intoxicated by larkspur. Research has shown that in steers, elevated heart rates, and muscle weakness can lead to recumbency coincides with large serum concentrations of larkspur alkaloids (9, 30, 50). Heart rates are typically elevated from 12 to 17 h after oral dosing (for example, upwards of 95 beats per minute after dosing with *D. barbeyi*) and then gradually return to normal for resting cattle over the following 76 h in laboratory studies (9, 40).

## Lupines (*Lupinus* spp.)

The genus *Lupinus* contains more than 500 species, both domesticated and wild. Lupines growing on rangelands, are deep-rooted, and high in protein making them attractive to grazing livestock (51). There are also domesticated lupines that include both ornamental and food cultivars, but they have lower concentrations of alkaloids. Problematic lupines representing multiple species growing on rangelands of Western North America can be both acutely toxic to livestock, and teratogenic to the developing fetuses of pregnant cattle. Acute lupine toxicity is due to the actions of piperidine and quinolizidine plant alkaloids at nAChR (52). Clinical signs of intoxication in adult cattle include discoordination and muscular weakness, exercise resistance, nictitating membranes partially covering the eyes, and death. Teratogenic lupines (lupines that contain the quinolizidine alkaloid, anagyrine, and / or select piperidine alkaloids) can cause fetal defects termed “crooked calf syndrome” in cattle (53–56). Crooked calf syndrome defects include: arthrogyrposis, kyposis, lordosis, scoliosis, torticollis, and cleft palate (57, 58). The mechanism behind the fetal defect formation is the inhibition of fetal movement by lupine alkaloid-induced receptor desensitization of fetal muscle-type nAChR (52, 59–61). Fetal muscle-type nAChR are quite sensitive to the desensitizing actions of these alkaloids, which profoundly inhibits fetal movement (59).

### Plant biomarkers

The biomarkers associated with lupine teratogenicity in cattle are the quinolizidine alkaloid anagyrine, and the piperidine alkaloids *N*-acetyl hystrine, ammodendrine, and *N*-methyl-ammodendrine (52, 53, 62–66). Quinolizidine alkaloids typically measured by gas chromatography mass spectrometry (GC-MS), and lupine alkaloid content has been found to be highly variable within and between plant species (54, 67–69). Of these alkaloids, the teratogen anagyrine is present in many lupines that have been associated with crooked calf syndrome, although its concentration can vary based upon environmental conditions, season, growth stage and lupine species (70, 71). This suggests that the teratogenic potential of western lupines changes from year to year due to differences in plant alkaloid concentrations. These observations explain the sporadic nature of crooked calf disease due to changes in the plant alkaloid concentrations because of the environmental conditions and other factors (55). Anagyrine has also been identified in extracts of blue cohosh (*Caulophyllum thalictroides*) a plant that is used as a human dietary supplement (72).

### Animal poisonings by lupines

Much like larkspur discussed above, toxic lupine species cause large economic losses from animal deaths, increased management costs, and reduced utilization of pastures and rangelands (55). Lupine alkaloids like anagyrine are rapidly absorbed in the rumen and appear in blood plasma just after oral dosing. Lupine alkaloids in a biological matrix like serum are typically measured with HPLC-MS (Table 2) (73–75). Lupine alkaloids are also rapidly eliminated from the serum of the bodies of cattle (30–35 h for 5 half-lives to pass). However, maternal serum and earwax anagyrine concentrations have been proposed for used as a biomarker for crooked calf disease (7, 76). In cattle, the most sensitive gestational period for exposure to lupine alkaloids like anagyrine is 40–70 days of gestation (63). Cattle breeds can respond differently to lupine alkaloid biomarkers during this period and, for example, when pregnant Holstein and Angus heifers were orally dosed with *L. leucophyllus*, fetal movements at eight and 12 h post dosing were significantly reduced in Angus heifers compared to Holstein heifers (Figure 5) (77).

**Table 2:** Serum toxicokinetic parameters of three alkaloids from *L. leucophyllus* orally dosed (2.5 g dried, ground plant material per kg body weight) to Holstein steers (73).

	**Anagyrine**	**Lupanine**	**5,6 -Dehydrolupanine**
E${}_{\frac{1}{2}}^{\text{a}}$, h	5.8 ± 0.6	7.0 ± 0.5	6.7 ± 0.3
D${}_{\frac{1}{2}}^{\text{b}}$, h	1.5 ± 0.9	5.0 ± 0.6	2.4 ± 1.3
A${}_{\frac{1}{2}}^{\text{c}}$, h	1.3 ± 0.6	5.4 ± 0.6	1.3 ± 0.7
Cmax^d^, ng/mL	2,761 ± 334	5,056 ± 1,094	632 ± 141
Tmax^e^, h	4.5 ± 1.0	15.5 ± 7.0	3.5 ± 0.6
AUC^f^, ng-h/mL	35,860 ± 7,395	162,427 ± 41,448	8,119 ± 2181

^a^E${}_{\frac{1}{2}}$, the elimination half-life.

^b^D_1/2_, the distribution half-life.

^c^A_1/2_, the absorption half-life.

d Cmax, describes the maximal serum alkaloid concentration.

e Tmax, describes the time of maximal serum alkaloid concentration.

^f^AUC, the area under the curve.

Angus heifers dosed with *L. leucophyllus*, had a significantly higher serum anagyrine concentrations when compared to Holstein heifer (*P* = 0.0076, 0.0026, and 0.0031 for 2, 4 and 8 h, respectively). The elimination half-life of anagyrine in Angus heifers was 5.6 ± 0.5 h compared to 3.7 ± 0.6 h in Holstein heifers (*P* = 0.0003, two-tailed *t*-test). This suggests that serum anagyrine concentrations can be used as a biomarker for crooked calf disease and that there are breed-dependent differences in the serum concentrations of lupine alkaloid biomarkers.

## Water hemlock (*Cicuta* spp.)

Water hemlocks (*Cicuta* spp.) are members of the carrot family (*Apiaceae*). There are four species of water hemlock that grow in the North America (78). These plants grow in wet habitats and are the most toxic plants found in North America (12). The tubers are more toxic than the vegetative parts of the plant (79, 80). Pharmacological studies indicate that the C_17_-polyacetylenes toxins in water hemlock act at GABA_A_ receptors to block the inhibitory signaling of the neurotransmitter GABA in the brain (49, 81). The actions of cicutoxin can readily be reversed by the benzodiazepine, diazepam *in vivo* and *in vitro* (49, 81). Water hemlock poisoning is somewhat unusual when compared to other North American toxic plants because clinical signs of intoxication are acute. Clinical signs of intoxication include frothy salivation, ataxia, dyspnea, muscular tremors, weakness, and violent terminal grand mal seizures followed by death. Animals that survive acute intoxication do not have any lasting effects. Death normally occurs within 1–8 h after consuming the plant (82).

### Plant and animal biomarkers for water hemlock poisoning

The bioactive compounds in water hemlocks are C_17_-polyacetylenes that include cicutoxin, cicutol, cicudiol, and isocicutoxin (83, 84). All parts of the plant are toxic, but the tubers have the largest concentrations of toxins, and thus they are most likely to poison livestock (80, 85). Recent research has also shown that toxin concentration as measured by HPLC with a photo diode array detector (HPLC-PDA) varies by geographic location suggesting that plant toxicity can vary depending on location and plant part (86). Cicutoxin and related toxins are unstable and readily oxidized in the rumen or stomach which limits their detection in biological samples from poisoned animals. This limits the use of cicutoxin as a biomarker for poisoning. Diagnosis of water hemlock poisoning can be made by documenting plant exposure from rumen or stomach contents. In surviving animals, elevated serum LDH, AST, and CK from seizure induced muscle damage peaks by 3 days after exposure then declines within 8–10 days after exposure (82).

## Swainsonine-containing plants

Swainsonine-containing plant poisoning was one of the first poisonous plant problems in Western North America and documented as early as 1,873. At that time, it was termed “locoism”, and the plants responsible “locoweeds”. Livestock losses due to swainsonine-containing plants are regional, sporadic, and can have significant economic impact (87).

**Figure 6:**
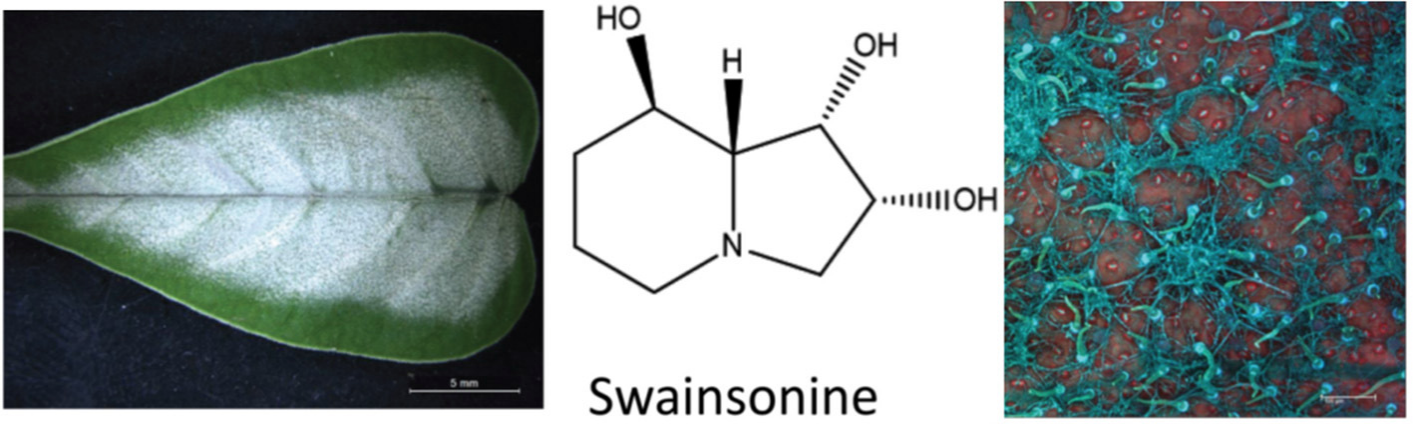
Ectopic growth of the Chaetothyriales fungal symbiont that produces swainsonine on *Ipomoea carnea* (93).

The plant alkaloid that causes locoism is swainsonine, an indolizidine alkaloid first discovered in *Swainsona canescens* by Colegate et al. (88). Subsequently, swainsonine has been reported in genera from three plant families, *Astragalus* and *Oxytropis*, Fabaceae; *Ipomoea* and *Turbina*, Convolvulaceae; and *Sida*, Malvaceae (89). Additional research has demonstrated that swainsonine is produced by fungal symbionts associated with the respective plant species that contain swainsonine (90–92). The presence of swainsonine as detected by HPLC-MS and the associated symbiotic fungi are biomarkers for plants that can cause lysosomal storage disease in grazing livestock (Figure 6). Swainsonine-containing species belonging to these three plant families have been documented in North America, South America, Africa, Australia, and China (94–97). Importantly, not all species in these genera contain swainsonine.

The mechanism of swainsonine toxicity is the inhibition of cellular α-mannosidases and as a result, the accumulation of oligosaccharides and glycoproteins in vacuoles of affected cells (neurons and hepatocytes) like that of a lysosomal storage disease (98). This cellular damage by swainsonine is the cause of neurological impairment manifested as proprioceptive deficits, emaciation, and reproductive dysfunction that is associated with livestock chronically consuming swainsonine-containing plants. The biochemical changes caused by chronic exposure to swainsonine can be used as a biomarker. For example, altered urinary glycoproteins as measured by thin layer chromatography (TLC) or HPLC, have been found in swainsonine treated goats, guinea pigs, rats, and sheep and are associated with the onset of clinical signs of poisoning (99–103). The presence of inhibited α-mannosidase can be used as a biomarker for ongoing chronic exposure to swainsonine. Nevertheless, mannosidase inhibition is transient and reversible once exposure to the toxin is stopped (104), and not very useful as a biomarker after exposure to the swainsonine-containing plants ends.

### Animal poisonings by swainsonine-containing plants

Swainsonine poisoning is most often a chronic exposure to swainsonine. For example, after 21 days of locoweed consumption, at 10–15% of the diet, estrus cycles in sheep are altered and conception rates reduced (105, 106). After 30 days of locoweed consumption, neurological deficits begin to manifest themselves and persist after exposure to swainsonine has stopped. As described above, clinical signs of poisoning include, weight loss, proprioceptive deficits, abortion in pregnant animals, and death. In range cattle grazing locoweed, there are changes in serum biochemistry with decreased α-mannosidase activity, and increased serum alkaline phosphatase, aspartate aminotransferase and lactate dehydrogenase (104). Similar changes in serum enzyme concentrations have been observed in sheep (107). The short half-life of swainsonine (elimination half-life of 20 h) prevents the usefulness of serum swainsonine concentrations as a biomarker. Histopathology is often the best diagnosis for locoweed poisoning. This is because of the microscopic lesions in the nervous and endocrine systems due to the accumulation of abnormally glycosylated proteins. Affected cells are filled with dilated vacuoles characteristic of a lysosomal storage disease (98, 107).

## Selenium-accumulating plants

Selenium (Se) is an essential micronutrient in livestock nutrition that has a relatively narrow window between deficiency and toxicity. Se deficiency can cause a nutritional myopathy (white muscle disease) that is more common than toxicity in livestock. Cases of Se toxicity occur when grazing seleniferous rangelands, contaminated pastures or when feed supplements or parenteral injections are mis-formulated. Clinical signs associated with acute Se poisoning include anorexia, depression, labored breathing, coma, and death. Chronic Se poisoning has been termed “alkali disease” and is characterized by hair loss, emaciation, and lameness (108).

### Seleniferous plants

Plants can be divided into three groups (Se-hyperaccumulators, facultative Se-accumulators, and passive Se-accumulators) based upon their ability to uptake and accumulate Se. Plants that can metabolize and store large concentrations (several thousand ppm dry weight) of Se predominantly as selenate and Se-methylselenocysteine are referred to as Se-hyperaccumulator plants and include but are not limited to many *Astragalus* spp., *Stanleya* spp., and *Aster* spp (109). These Se-hyperaccumulator plants are also referred to as Se-indicator or obligate Se-accumulator plants because they are most often found growing on soils high in bioavailable Se; therefore, they can be helpful in locating and identifying areas with plants containing elevated Se concentrations that are a risk to poison livestock. Plants that can thrive both on seleniferous and normal soils are referred to as facultative Se-accumulators can accumulate from several hundred to two thousand ppm Se. Passive Se-accumulator plants can contain 5 to ~150 ppm Se on some Se-containing soils but are often stressed or eliminated as soil Se concentrations become elevated. It is the plants that contain 5–150 ppm Se that cause most subacute or chronic toxicity problems for livestock. Palatability decreases as plant Se concentration increases (110) and as plant Se concentration exceeds ~25 to 30 ppm livestock will prefer to avoid them unless other forages are unavailable.

### Animal biomarkers of selenium poisoning

**Figure 7:**
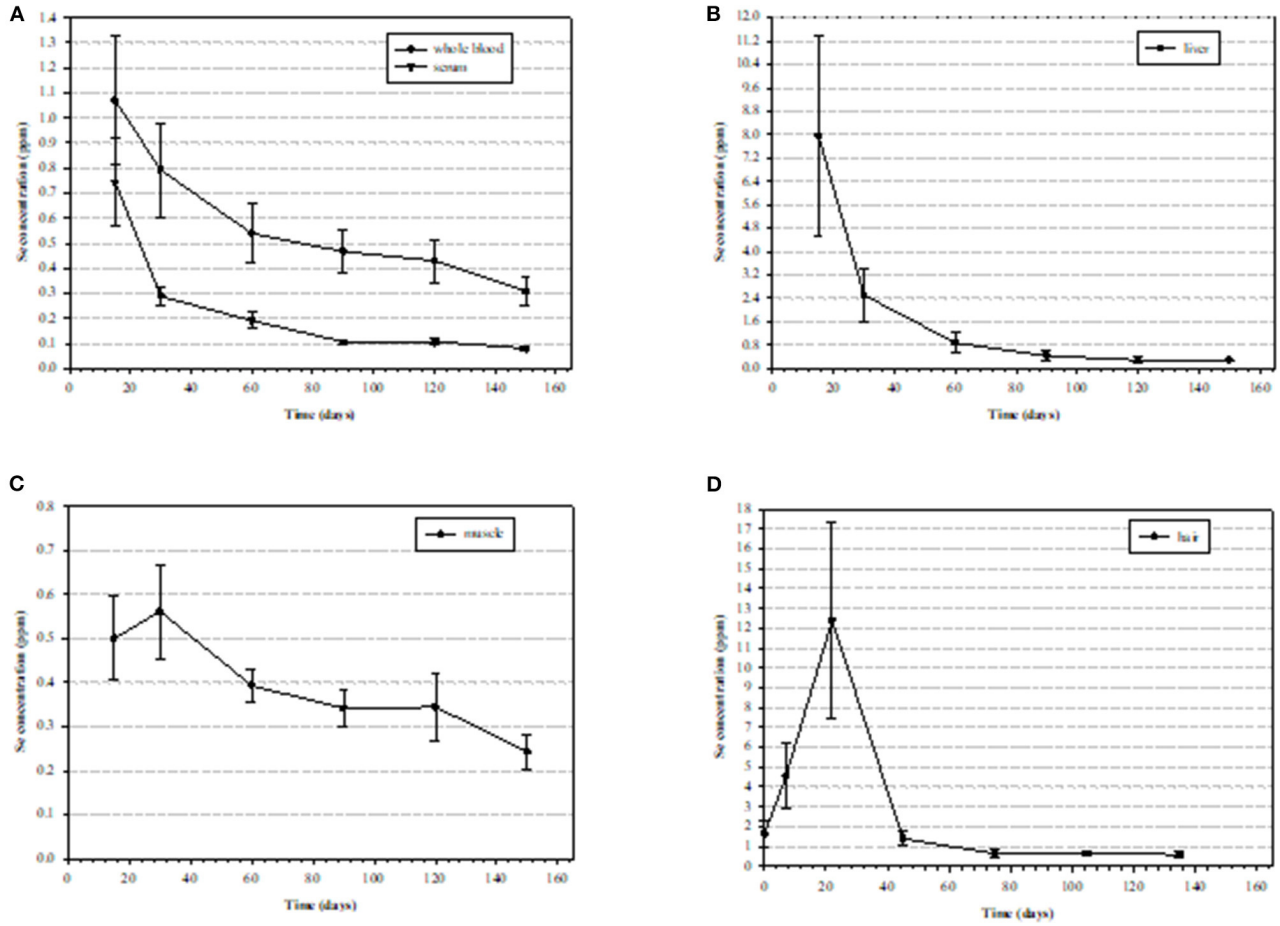
Mean (± standard deviation [SD]) selenium (Se) concentrations (ppm) in tissues of nine steers starting at 15 days post exposure to high Se-containing western aster in **(A)** whole blood with an elimination half-liver (E_1/2_) of 115.6 ± 25.1 days and serum with and E_1/2_ of 40.5 ± 8.2 days), **(B)** liver (wet weight) with an E_1/2_ of 38.2 ± 5.0 days, **(C)** semitendinosus muscle E_1/2_ with an E_1/2_ of 98.5 ± 19.1 days, **(D)** hair (dry weight) (111).

**Figure 8:**
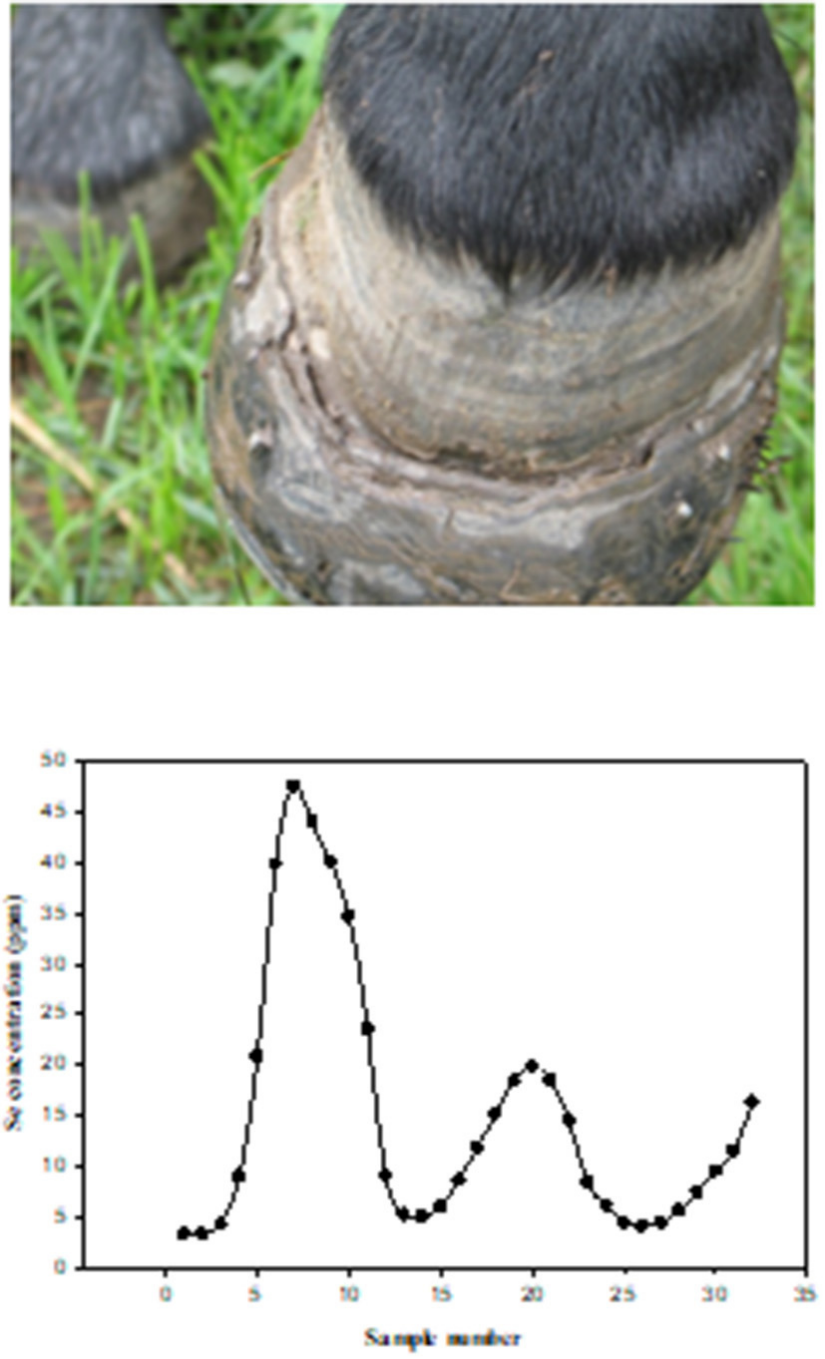
Photograph of left front hoof of a horse chronically poisoned on selenium, seven months after being removed from the pasture containing high selenium forage and water. Figure 2B. Selenium (Se) concentrations in tail segments (2.5 cm), from a horse that grazed in a pasture with high Se forages and water. The hair segments were numbered sequentially with the most recent growth (closest to skin) being number 1. The horse grazed in the pasture from approximately May 15th to November 15th for three consecutive years (114).

When diagnosing Se status or exposure of livestock it is important to examine both the animal and its environment. A complete health history of mineral supplementation is needed, as well as feed and forage consumption, in addition to clinical signs which provide the basis for suspected Se poisoning (acute or chronic). Samples are most often analyzed for Se by inductively coupled plasma-mass spectrometry (ICP-MS) and include blood, serum, kidney, liver, urine, rumen contents, hoof, and/or hair. Each sample has its advantages and disadvantages for analysis depending upon species, potential exposure history, time elapsed post-exposure, and condition of the animal. Se, in general, is slowly excreted, requiring relatively long withdrawal times (weeks) (3, 111). Normally, kidney Se concentrations are greater than liver Se concentrations, however, in cases of Se poisoning the concentrations can be reversed. Clinical signs of acute Se poisoning are usually observed within 6–24 h post-exposure while signs of chronic poisoning may not be apparent for several months. Diagnosing lethal acute Se poisoning is most accurately done be determining liver Se concentrations by ICP-MS. Normal liver Se concentrations for most livestock species are in the range of 0.25–0.5 ppm (wet weight, w.w.) while acutely poisoned animals will often exceed 5 ppm Se, w.w. (112). Confirming or diagnosing sublethal acute Se poisoning is usually done by whole blood Se analysis or with a liver sample collected by a liver biopsy (Figure 7). Since Se is slowly excreted, samples can be collected several weeks post-exposure if necessary. Additionally, increases in serum creatine kinase and troponin I can also be used as biomarkers of acute selenosis to support other data due to the muscle damage caused by abnormal Se concentrations (113). Hair samples can also be analyzed to determine previous Se exposure. In cattle a hair sample can be clipped from the shoulder area close to the skin to determine Se content several weeks post-exposure. Approximate time of previous Se exposure (possibly up to 1–3 years previously) can be estimated by collecting mane or tail hair of horses (Figure 8) (114) or hair from a tail switch of cattle and then segmenting the hair and analyzing each segment for Se content while estimating growth rates. A similar approach to determining time and extent of exposure can be done by fragmenting and analyzing hooves for Se content (115).

Acute Se poisoning is usually caused because of over-supplementation or overdosing with parenteral preparations or due to animals (most often naïve animals) consuming a toxic dose of Se-accumulator plants. It has been reported that acute oral Se poisoning occurs with sudden exposure of Se at 2.2 to ~20 mg Se/kg (body weight, b.w.) depending upon age, species and form or Se (109, 116). Oftentimes, if livestock ingest a significant but nonlethal dose, they will become depressed, go off feed for 1–3 days and then they will avoid elevated Se forages in the future (110). Acutely poisoned animals will often present with signs of depression, reduced food intake and tachypnea 6–18 h post-exposure. Sheep and cattle usually die due to myocardial lesions, pulmonary edema, and hemorrhage (111, 117). Gross pathological findings are usually limited to pulmonary congestion and accumulation of edema, and myocardial necrosis with pale streaks and hemorrhagic areas of the myocardium with severity often depending upon time of death post exposure. Animals that survive more than 72 h post-exposure will most likely recover, however, some individuals may suffer from myocardial necrosis with subsequent heart failure seen as passive congestion as well as centrilobular hepatic congestion with hepatocellular degeneration and necrosis (114).

Alkali disease is a result of chronic (usually >30 days) Se exposure to forages or feeds that have elevated Se concentrations (usually 5–30 ppm). The most obvious clinical signs of chronic poisoning include alopecia (in many but not all cases) and symmetric dystrophic hoof growth which results in lameness and the inability to travel to feed and water resulting in emaciation. Chronically poisoned animals can also develop heart failure with both necrosis and fibrosis in the myocardium. If early signs of lameness, swelling of the coronary bands and hair loss are noticed resulting in removal of the elevated Se sources some of the more severe issues of lameness can be avoided. Horses are very susceptible to chronic Se poisoning with sheep being much more resistant. Raisbeck (115) had suggested that, when comparing chronic Se poisoning in livestock, the order from most sensitive to least sensitive is swine (most sensitive) > horses > cattle >sheep (least sensitive). The treatments for chronic Se poisoning are limited but removal of the source of exposure is necessary along with a low Se, high quality and high protein diet with balanced micronutrients. When hoof lesions are observable supportive hoof care with pain control, therapeutic trimming and shoeing are necessary.

## Dehydro-pyrrolizidine alkaloid containing plants

**Figure 9:**
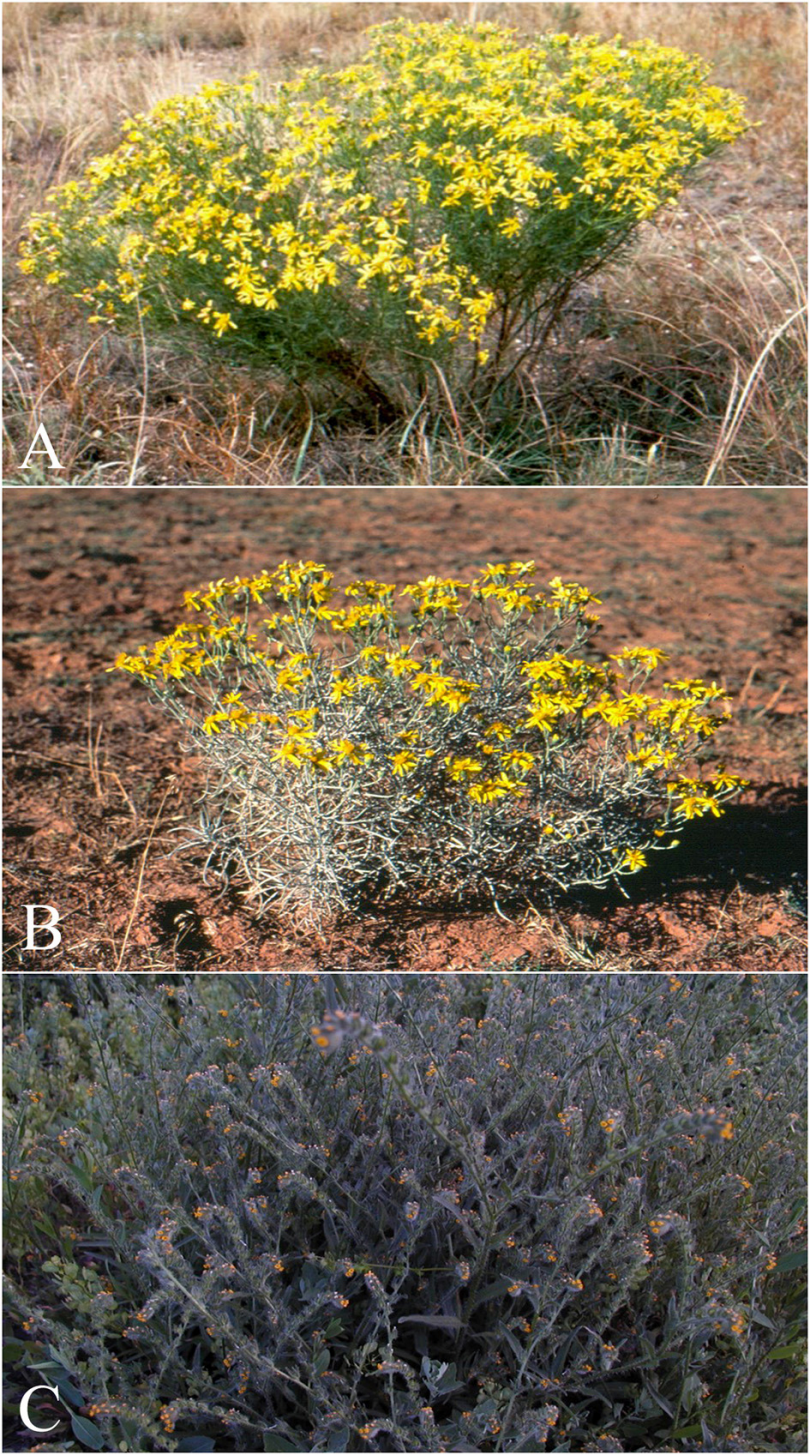
Dehydro-pyrrolizidine alkaloid (PA) containing plants that commonly poison livestock in North America. **(A)** *Senecio riddellii* is found in the central United States and most commonly poisons cattle. This plant is unique, in that it primarily contains a single dehydropyrrolizidine alkaloid (PA), riddelliine, whereas most PA containing plants contain mixtures of various PAs. Riddelliine has been proven carcinogenic to rodents and is classified as a potential human carcinogen by the national toxicology program. **(B)** *Senecia douglasi* var. longilobus (threadleaf or wooly groundsel) is found in several southwestern states. The plant contains four different PAs, and most commonly poisons cattle. Wooly groundsel grows well on abused or arid rangelands. And **(C)** *Amsinckia intermedia*, (tarweed or fiddleneck) is found throughout Canada and the western, midwestern and northeastern United States, and poisons cattle and horses. This plant has been reported to cause the clinical syndromes “walking disease” in horses and “hard liver disease” in cattle. It commonly grows in waste areas and along field margins.

Dehydro-pyrrolizidine alkaloid (PA) containing plants occur throughout the world and it has been estimated that they compose nearly 5% of flowering plants. However, not all PA containing plants result in poisoning, only about 30 species of the Boraginaceae, Asteraceae, Orchidaceae, and Fabaceae families have been reported to poison livestock, wildlife, and humans (Figure 9). Toxicity varies with the specific plant, alkaloid(s) and animal species poisoned. For example, pigs and chicks are highly sensitive to poisoning, while goats and mice are resistant to PA toxicity. Most PAs are hepatotoxic, others are pneumotoxic or nephrotoxic, and still others have been shown to be carcinogenic. Many toxic PA-containing plants are noxious weeds that invade fields, pastures and rangelands, most poisonings are a result of PA contamination of feed and food. Most clinical disease results from chronic intoxication that may not become clinical for weeks or even months after exposure. This often complicates diagnosis because the contaminated food or feed may not be available for examination or analysis. Most PA containing plants are not palatable, animals must be forced to eat them when alternative forages are not available. When measured by HPLC-MS, the concentrations of PAs in plant populations are also highly variable even in the same plant and location from year to year (47, 118). This variation makes it difficult to predict toxicity; calculate toxic doses when ingestion is suspected; and develop risk-based management strategies to minimize or avoid poisoning.

PAs require bioactivation *via* hepatic cytochrome P450 enzymes to be toxic. The resulting electrophiles quickly react with adjacent proteins, nucleic acids, and membranes resulting in hepatic damage and subsequent liver disease. As with many tissues, the liver has limited methods and responses to damage and consequently many genetic, nutritional, infectious, and toxic disease produce similar clinical, gross, and microscopic lesions. Many PA metabolites quickly bind nucleic acids and have an antimitotic effects that can result in megalocytosis, a characteristic histologic finding in which hepatocytes and their nuclei become enlarged due to the inability to divide. There is evidence that protein or DNA adducts persist in tissues for months to years and may be recycled to cause additional damage. The objectives of this review are to present biomarkers of PA-associated disease and how those relate to the diagnosis and prognosis of poisoning.

### Clinical PA-induced disease

At high doses poisoned animals commonly display clinical signs of acute liver failure, including anorexia, depression, icterus, visceral edema, and ascites. Biochemical changes of liver damage and loss of liver function include increased aspartate aminotransferase (AST), succinate dehydrogenase (SDH), alkaline phosphatase (ALP), and gamma-glutamyl transferase (GGT) activities. These are followed by markers of impaired hepatic function or metabolism including increased concentrations of bilirubin, bile acids and ammonia. Later functional indicators of hepatic metabolism such as blood urea nitrogen (BUN), total protein, albumin and certain coagulation proteins may decrease. If the duration is long enough, poisoned animals may develop altered chlorophyll metabolism, hyperammonemia, intense icterus, and coagulopathies. These changes clinically present as photosensitivity, neurologic disease, edema, hemorrhages, other coagulopathies including thrombosis and infarctions.

Chronic poisoning is usually associated with low doses that may occur sporadically or intermittently. Such exposures often occur with contaminated forages as weed infestation and subsequent contamination are patchy. The resulting clinical disease often requires weeks or months to become clinical or observable. The resulting periodic or transitory disease may include clinical malaise, followed by recovery times with minimal clinical change. Liver serum enzyme activities (AST, SDH, ALP, and GGT) may be temporarily elevated; animals may become anorexic and develop liver failure. If the damage is moderate, biochemical changes might include elevated enzymes, hyperbilirubinemia, and bile acids, and decreased indicators of hepatic metabolism (BUN, Pro, and Alb). Some animals completely recover while others are permanently damaged. These animals may temporarily compensate and appear to recover, but they relapse when stressed with pregnancy, lactation, or a hard winter. Such recurrent liver disease may include icterus, hyperbilirubinemia, altered chlorophyll metabolism with subsequent photosensitization. These animals often progress and develop end stage liver disease (cirrhosis) with biochemical and clinical changes relative to the degree of loss of liver function (47).

### Microscopic changes of PA poisoning

**Figure 10:**
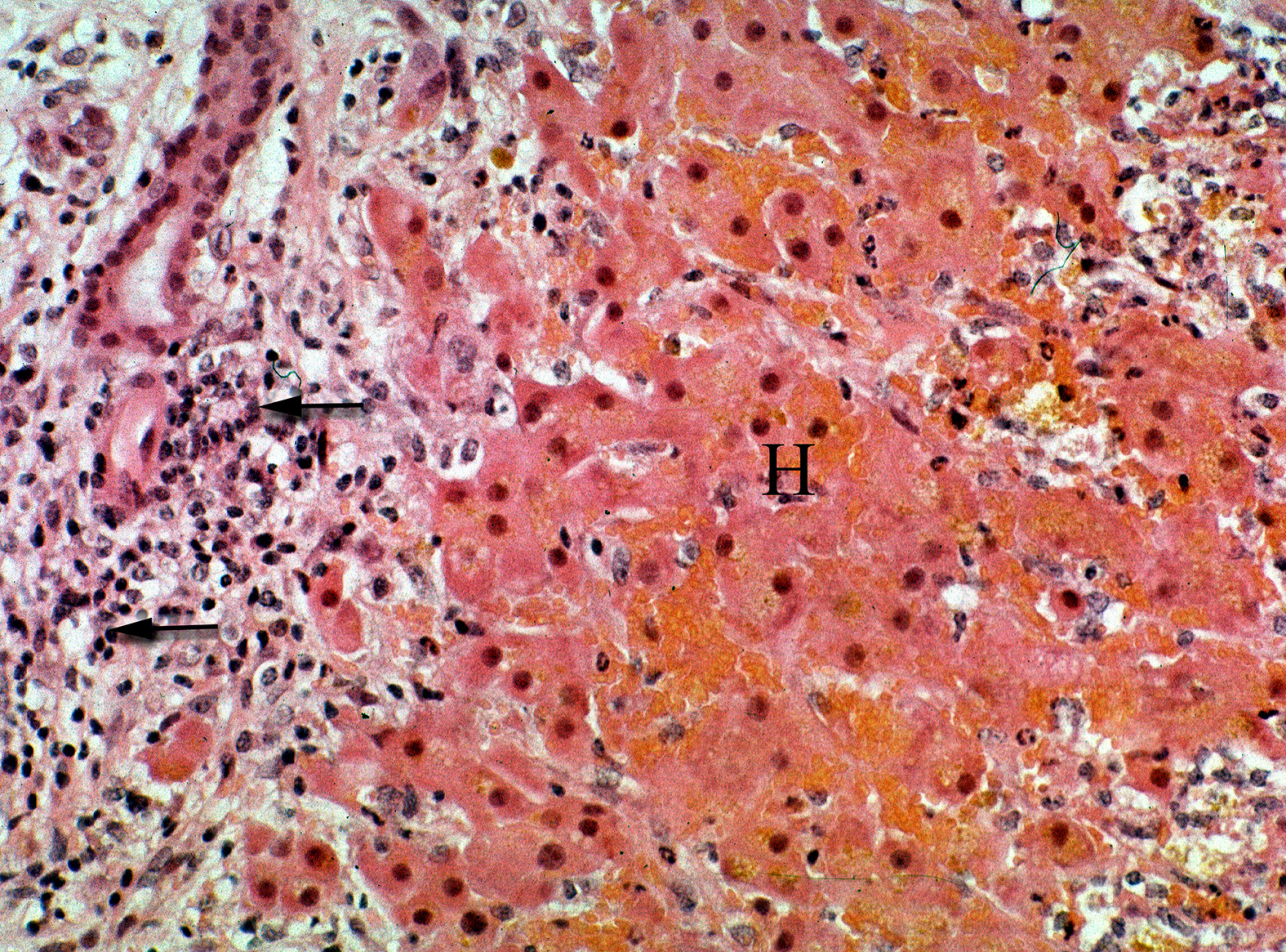
Photomicrograph of a liver from horse treated with ground, dried hounds tongue (*Cynoglossum officinale*) to obtain a dose of 15 mg total dehydropyrrolizidine alkaloids/kg BW for 9 days. The horse developed liver failure and was euthanized and necropsied. Notice the focally extensive hemorrhagic necrosis (H) that is characterized by the replacement of hepatic cords with erythrocytes and cellular debris. This reaction is both centrilobular and midzonal. No central vein is seen in this section. The periportal region is infiltrated with numerous lymphocytes mixed with proliferating ovalocytes and fibroblasts. Some ovalocytes are beginning to form rows and small tubules (biliary proliferation). Figure modified from previous publication (119).

Microscopic changes associated with PA poisoning are characteristic, but not specific for PA-induced damage. Other toxins such as alkylating toxins like aflatoxins often produce similar microscopic changes. Acute poisoning generally involves high doses of relatively short exposures. This results in acute hepatocellular swelling with degeneration that quickly progresses to necrosis. Though the distribution of necrosis is alkaloid specific, with high doses they all progress to pan lobular necrosis. This lesion may be characterized as hemorrhagic necrosis as pools of erythrocytes that replace normal hepatic cords (Figure 10). This pan-lobular necrosis is characterized by fragmentation and loss of hepatocytes and rare swelling and degeneration of periportal hepatocytes with minimal inflammation. Such massive necrosis is generally associated with doses that are uncommon in clinical poisonings. Severe necrosis could be caused by aflatoxin, *Xanthium strumarium* (cocklebur) or *Salvia reflexa* (wild mint) it is fortuitous that such PA-induced necrosis nearly always has ample amounts of detectable PA metabolites (pyrroles that are discussed later).

**Figure 11:**
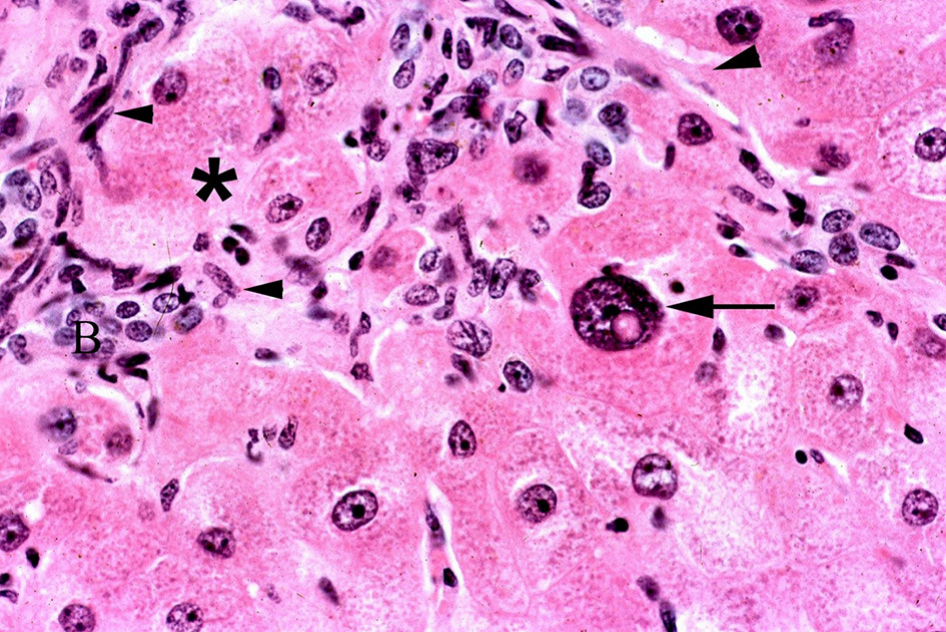
Photomicrograph of a liver biopsy from horse treated with ground, dried hounds tongue (*Cynoglossum officinale)* to obtain a dose of 5 mg total dehydropyrrolizidine alkaloids/kg BW for 10 days and allowed to recover for 45 days. Notice the fibroblast proliferation that entraps hepatocytes that are swollen and degenerative (*). One large megalocyte is also present with a large nucleus and prominent nuclear vacuole (arrow). There is also oval cell proliferation (arrowheads) and increased small biliary structures. Figure modified from previous publication (119).

Chronic intoxication is characterized by individual hepatocyte degeneration and necrosis. Like acute poisoning, these lesions may begin with centrilobular or midzonal distribution; however, this distribution is obscured by secondary changes of fibrosis and biliary hyperplasia (Figure 11). This triad of necrosis, fibrosis and bile duct proliferation are generally recognized as typical or characteristic of PA poisoning. However, this also is not pathognomonic as other diseases especially aflatoxicosis produce similar changes. Regardless of the initial distribution with time the lesions become periportal with extensive fibrosis, oval cell proliferation and biliary hyperplasia and necrosis. The necrosis often affects hepatocytes that become surrounded or entrapped by proliferative connective tissue. Some PAs are anti-mitotic resulting in large nuclei or hepatic megalocytes (47, 120).

### Chemical identification of PA and PA metabolites

**Figure 12:**
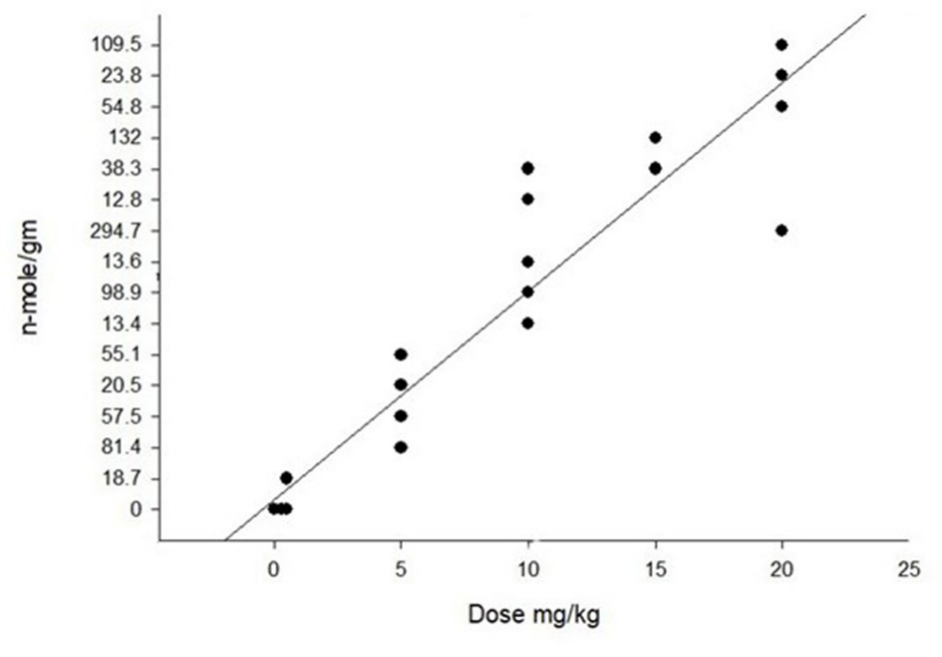
Graph of pyrrole (dehydropyrrolizidine alkaloid metabolite) concentration in the liver of pigs that were dosed with purified riddelliine. As seen with the regression there is a positive correlation with dose that was significant with *r*^2^ = 0.90. Pyrrole analysis was done following previously described techniques (4). Similar positive correlations of pyrrole concentrations with PA dose have been seen in other species with variations relating duration and recovery time. Modification of previously reported data (122).

PA detection in rumen contents, serum or tissues has limited usefulness as they are quickly excreted or metabolized. The metabolites or pyrroles quickly react with tissues, especially liver tissues. Improvements in chemical detection of these pyrroles using LC MS/MS have not only made detection more dependable but also has made it possible to quantitate their concentrations (121). The amount of tissue bound pyrroles is dependent on alkaloids, dose, duration, and clearance time. Using saved liver samples from PA studies in cattle, horses, pigs, chickens, and rodents we found a positive correlation between dose and liver pyrrole concentration (Figure 12). We also found that different PAs produce different amounts of pyrroles. For example, riddelliine often produces nearly 10 times more liver pyrrole concentrations than other similarly toxic alkaloids such as senecionine, and seneciphylline (121, 123).

### Interpretation and diagnosis

Identification of PA poisoning is often challenging. Clinical signs may be delayed for weeks and even months after ingestion. Because many intoxications are caused by contaminated feeds, the clinical disease may not become apparent until after the contaminated feed is consumed and unavailable for examination. Serum biochemical changes are good indicators and biomarkers of non-specific liver damage (increased AST, ALP, SDH, GGT) and decreased liver function (increased bilirubin and decreased BUN, Pro, Alb and coagulation proteins). If PA poisoning is suspected, rumen contents and liver samples should be collected and stored frozen. If gross and microscopic findings support PA intoxication these samples can be analyzed for more specific biomarkers (PAs and PA metabolites or pyrroles) (121). Pyrrole detection provides definitive evidence of both exposure and consumption, but it does not indicate PAs are the cause of either disease or death (121). False positives do occur when pyrroles are detected in clinically and microscopically normal animals. This is most common when relatively resistant species like rodents and small ruminants ingest PA containing plants. However, when a positive pyrrole result is combined with supporting clinical signs and histologic lesions consistent with PA poisoning, the diagnosis becomes the most likely cause. However, there is no pyrrole concentration that definitively indicates PA toxicity and disease. This is due to marked variability of animal susceptibility to DHPA poisoning, variability of individual alkaloid toxicity, and PA specific pyrrole production.

## Conclusions

Poisonings by toxic plants occurs when animals eat too much, too fast, or graze them over extended periods of time. This article reviewed biomarkers of plant poisoning in grazing livestock in the Western North America. Methods of noninvasive sampling such as earwax, hair and saliva were presented. These methods of sampling livestock provide a less invasive means to obtain biological samples than venipuncture in live animals and the rumen contents of dead animals which has been traditionally used (Table 3). Plants discussed include larkspur, lupine, water hemlock, swainsonine-containing plants, selenium-containing plants, and pyrrolizidine alkaloid containing plants. These six plants are most often associated with both acute and chronic poisonings in Western North America and cause significant economic losses (2).

**Table 3:** Poisonous plants of Western North America, biomarkers, affected species, clinical signs, detection, and sampling.

**Plant**	**Biomarkers**	**Species, clinical signs**	**Biomarker detection**	**Noninvasive sampling**
Larkspur (*Delphinium* spp.)	The *N*-(methylsuccinimido) anthranoyllycoctonine type alkaloids like methyllycaconitine, and 7,8-methylenedioxylycoctonine type like deltaline (24–26).	Cattle [heifers are especially susceptible (48)], depression, muscle weakness, recumbency, bloat, elevated heart rate and death.	HPLC-MS, FTIR	Yes, detected in earwax, oral fluids, nasal mucus (8).
Lupine (*Lupinus* spp.)	The quinolizidine alkaloid anagyrine, and the piperidine alkaloids *N*-acetyl hystrine, ammodendrine, and *N*-methyl-ammodendrine (52, 53, 62–66).	Cattle, sheep and goats. Discoordination and muscular weakness, exercise resistance, nictitating membranes partially covering the eyes, and death (53–56).	In plant material, GC-FID and GC-MS. In animal samples, HPLC-MS.	Yes, detected in earwax, hair, oral fluid, and nasal mucus (7).
Water Hemlock (*Cicuta* spp.)	C17-polyacetylenes, cicutoxin, cicutol, cicudiol, and isocicutoxin (83, 84).	All animals, frothy salivation, ataxia, dyspnea, muscular tremors, weakness, and violent terminal grand mal seizures followed by death.	HPLC with PDA detection in plant material.	No, C17-polyacetylenes are rapidly oxidized in rumen or stomach.
Swainsonine containing plants. (*Astragalus, Oxytropis*, and other spp.)	Swainsonine which is produced by fungal symbionts of the plant host (90–92).	Grazing livestock, proprioceptive deficits, emaciation, and reproductive dysfunction that is associated with chronic consumption of swainsonine-containing plants.	In plant material GC-MS and HPLC-MS, in serum HPLC-MS. Urine by TLC and HPLC.	Yes, altered urinary glycoproteins (99–103).
Selenium-accumulators, *Astragalus, Stanleya*, and *Aster* spp (109).	Se toxicity occurs on seleniferous rangelands, contaminated pastures or when feed supplements or parenteral injections are mis-formulated.	Grazing livestock, anorexia, depression, labored breathing, coma, and death. Chronic Se poisoning has been termed “alkali disease” and is characterized by hair loss, emaciation, and lameness (108).	ICP-MS	Yes, hooves, hair (3).
Dehydro-pyrrolizidine alkaloid (PA) containing plants Boraginaceae, Asteraceae, Orchidaceae, and Fabaceae families	Selected PAs including; heliotrine, lasciocarpine, riddelliine, senecionine, and seneciphylline (121, 123).	All livestock, anorexia, depression, icterus, visceral edema, and ascites biomarkers include non-specific liver damage (increased AST, ALP, SDH, GGT) and decreased liver function (increased bilirubin and decreased BUN, Pro, Alb, and coagulation proteins.	HPLC-MS, PA metabolites (pyrroles) can be measured. There is no pyrrole concentration that definitively indicates PA toxicity and disease.	No, If PA poisoning is suspected, rumen contents and liver samples can be obtained (122).

When diagnosing plant poisonings of livestock, it is imperative that the main elements of plant poisoning be considered: the plant must be present in the environment; the toxin must be present in the plant material; the toxin is present in biological samples from the intoxicated animal(s); clinical signs consistent with poisoning of the suspected plant were observed; and less specific biomarker(s) of poisoning such as hematology, serum biochemical, or tissue lesions are consistent with the suspected poisonous plant. These elements of poisoning can vary with livestock species There are many other poisonous plants which require further work to better describe clinical signs of poisoning and plant and animal biomarkers.

## Author contributions

All authors contributed to the creation of this review article, refer to the reference list for details about specific original research contributions.

## Footnotes

^1^ https://www.cancer.gov/publications/dictionaries/cancer-terms/def/biomarker
